# Microvascular blood flow velocities measured with a retinal function imager: inter-eye correlations in healthy controls and an exploration in multiple sclerosis

**DOI:** 10.1186/s40662-018-0123-0

**Published:** 2018-11-02

**Authors:** Liang Wang, Ohemaa Kwakyi, James Nguyen, Esther Ogbuokiri, Olwen Murphy, Natalia Gonzalez Caldito, Laura Balcer, Elliot Frohman, Teresa Frohman, Peter A. Calabresi, Shiv Saidha

**Affiliations:** 1 Department of Neurology, Johns Hopkins University School of Medicine, Baltimore, MD USA; 2 Departments of Neurology, Population Health and Ophthalmology, New York University School of Medicine, New York, NY USA; 3 Departments of Neurology and Ophthalmology, University of Texas Austin Dell Medical School, Austin, TX USA

**Keywords:** Inter-eye correlation, Blood flow velocity, Retinal function imager, Neurology, Multiple sclerosis, Optic neuropathy

## Abstract

**Background:**

The retinal microcirculation has been studied in various diseases including multiple sclerosis (MS). However, inter-eye correlations and potential differences of the retinal blood flow velocity (BFV) remain largely unstudied but may be important in guiding eye selection as well as the design and interpretation of studies assessing or utilizing retinal BFV. The primary aim of this study was to determine inter-eye correlations in BFVs in healthy controls (HCs). Since prior studies raise the possibility of reduced BFV in MS eyes, a secondary aim was to compare retinal BFVs between MS eyes, grouped based on optic neuritis (ON) history and HC eyes.

**Methods:**

Macular arteriole and venule BFVs were determined using a retinal function imager (RFI) in both eyes of 20 HCs. One eye from a total of 38 MS patients comprising 13 eyes with ON (MSON) and 25 eyes without ON (MSNON) history were similarly imaged with RFI.

**Results:**

OD (right) and OS (left) BFVs were not significantly different in arterioles (OD: 3.95 ± 0.59 mm/s; OS: 4.08 ± 0.60 mm/s, *P* = 0.10) or venules (OD: 3.11 ± 0.46 mm/s; OS: 3.23 ± 0.52 mm/s, *P* = 0.06) in HCs. Very strong inter-eye correlations were also found between arteriolar (*r* = 0.84, *P* < 0.001) and venular (*r* = 0.87, *P* < 0.001) BFVs in HCs. Arteriolar (3.48 ± 0.88 mm/s) and venular (2.75 ± 0.53 mm/s) BFVs in MSNON eyes were significantly lower than in HC eyes (*P* = 0.009 and *P* = 0.005, respectively). Similarly, arteriolar (3.59 ± 0.69 mm/s) and venular (2.80 ± 0.45 mm/s) BFVs in MSON eyes were also significantly lower than in HC eyes (*P* = 0.046 and *P* = 0.048, respectively). Arteriolar and venular BFVs in MSON and MSNON eyes did not differ from each other (*P* = 0.42 and *P* = 0.48, respectively).

**Conclusions:**

Inter-eye arteriolar and venular BFVs do not differ significantly in HCs and are strongly correlated. Our findings support prior observations that arteriolar and venular BFVs may be reduced in MS eyes. Moreover, this seems to be the case in both MS eyes with and without a history of ON, raising the possibility of global blood flow alterations in MS. Future larger studies are needed to assess differences in BFVs between MSON and MSNON eyes.

## Background

Using the retinal microvasculature as an indirect surrogate of the systemic, and in particular cerebral microvasculature, is a long-standing concept [1, 2]. As such, the initial intent of fluorescein angiography was for this purpose, but its invasive nature and potential for adverse events limited its widespread utility [3]. Despite numerous advances across multiple imaging modalities over the years, there has been limited advancement in imaging the cerebral microcirculation even with highly specialized brain imaging techniques. Since the retina is an extension of the brain, cerebral and retinal tissues share multiple common embryologic features, making the homologous retina potentially ideal for making inferences about the cerebral microvasculature. With the advent of new imaging techniques including the retinal function imager (RFI) and spectral domain-optical coherence tomography angiography (OCT-A), there has been a renewed interest in studying the retinal microcirculation as a surrogate of cerebral microcirculation in neurologic diseases [4].

Direct measurement of retinal blood flow velocity (BFV) may provide important information about the cerebral microcirculation [1, 2] and also systemic circulation [3, 4]. Retinal BFV measured in a relatively wide field of view using the retinal function imager (RFI) has been used to characterize the retinal microcirculation in previous studies of ocular, systemic and cerebral diseases, including age-related macular degeneration [5], diabetes [6], and multiple sclerosis (MS) [2]. Such studies have helped to contribute to our understanding of disease onset, progression, and underlying vasculopathogenesis in these disorders. The repeatability and reproducibility of RFI-derived measurements from one eye of each subject have been reported in previous studies. As such, the measurement of retinal BFVs with RFI has been shown to be reproducible with approximately 10% variability [7]. Although differences in RFI measures have been assessed between eyes in various disease states and healthy control (HC) eyes, it remains to be determined whether BFVs vary significantly between OD (right) and OS (left) eyes in HCs. This is important for helping to guide the interpretation of prior study findings as well as the design, including eye selection, of future studies assessing or utilizing retinal BFV.

Multiple sclerosis (MS) is a chronic, inflammatory, demyelinating disorder, in which neurodegeneration is thought to be the principal substrate underlying disability. MS primarily affects young and middle-aged adults and is the most common non-traumatic cause of neurologic disability in the developed world. Despite advances in our understanding of the pathobiology of MS, the etiology and pathologic mechanisms of this complex neurological disorder remain unclear. MS has many characteristics that may be associated with vascular alterations, such as inflammatory cerebral endotheliopathy [8], vessel occlusion [9], vascular wall thickening, enhanced deposition of perivascular collagen, blood-brain barrier disruption [10], and perivascular inflammation. These vascular processes may contribute to the diffuse hypoperfusion that has been suggested to occur in the normal-appearing white and gray matter of MS patients [11–15]. Decreased perfusion could theoretically impair tissue oxygenation [16] and contribute to neurodegeneration in MS, although this remains to be elucidated, and thus it is also plausible that decreased perfusion may simply be a secondary consequence of reduced oxygen demand in areas of neurodegeneration. Considering that, several histopathological studies insinuate hypoxia-like tissue injury in MS lesions [17–19]. Indeed, increases in hypoxia inducible factors (implying vascular compromise) have been found in regions of neurodegeneration in MS. A recent cross-sectional study of RFI in relapsing remitting MS (RRMS) revealed reductions in retinal arteriolar and venular BFVs when compared to HCs [2]. This study was however small (*n* = 17), and therefore did not allow for exploration of differences in BFVs between MS eyes with and without a history of optic neuritis (ON), as compared to HC eyes [2]. Moreover, while the study findings are supported by recent OCT-A studies in MS, they remain to be confirmed [20, 21]. Furthermore, comparisons in this study did not randomly select between OD and OS, which may introduce a bias if in fact there are inter-eye differences in BFVs.

The primary aim of this study was to determine inter-eye correlations in BFVs in healthy controls (HCs). Our secondary aim was to confirm that BFVs are reduced in MS eyes and compare retinal BFVs between MS eyes grouped by ON history and HC eyes.

## Methods

### Participants

We recruited 20 HCs from Johns Hopkins University staff and 38 MS patients from the Johns Hopkins Multiple Sclerosis Center. The study was approved by the Institutional Review Board of Johns Hopkins University and written informed consent was obtained from all study participants. MS diagnosis was confirmed by the treating neurologist following the 2010 revised McDonald criteria [22]. Patients with acute ON within 6 months of assessment and any subjects with refractive errors of greater than +/− 6 diopters, history of ocular surgery, glaucoma, hypertension, diabetes or other neurological or ophthalmological pathology were excluded from the study. Subjects that had less than four series with four in-focus images were excluded for quality control purposes as recommended [23–25]. In total, seven out of 27 (leaving 20) HCs and 18 out of 56 (leaving 38) MS patients were excluded due to an insufficient number of usable images in either or both eyes.

### RFI

**Fig. 1:**
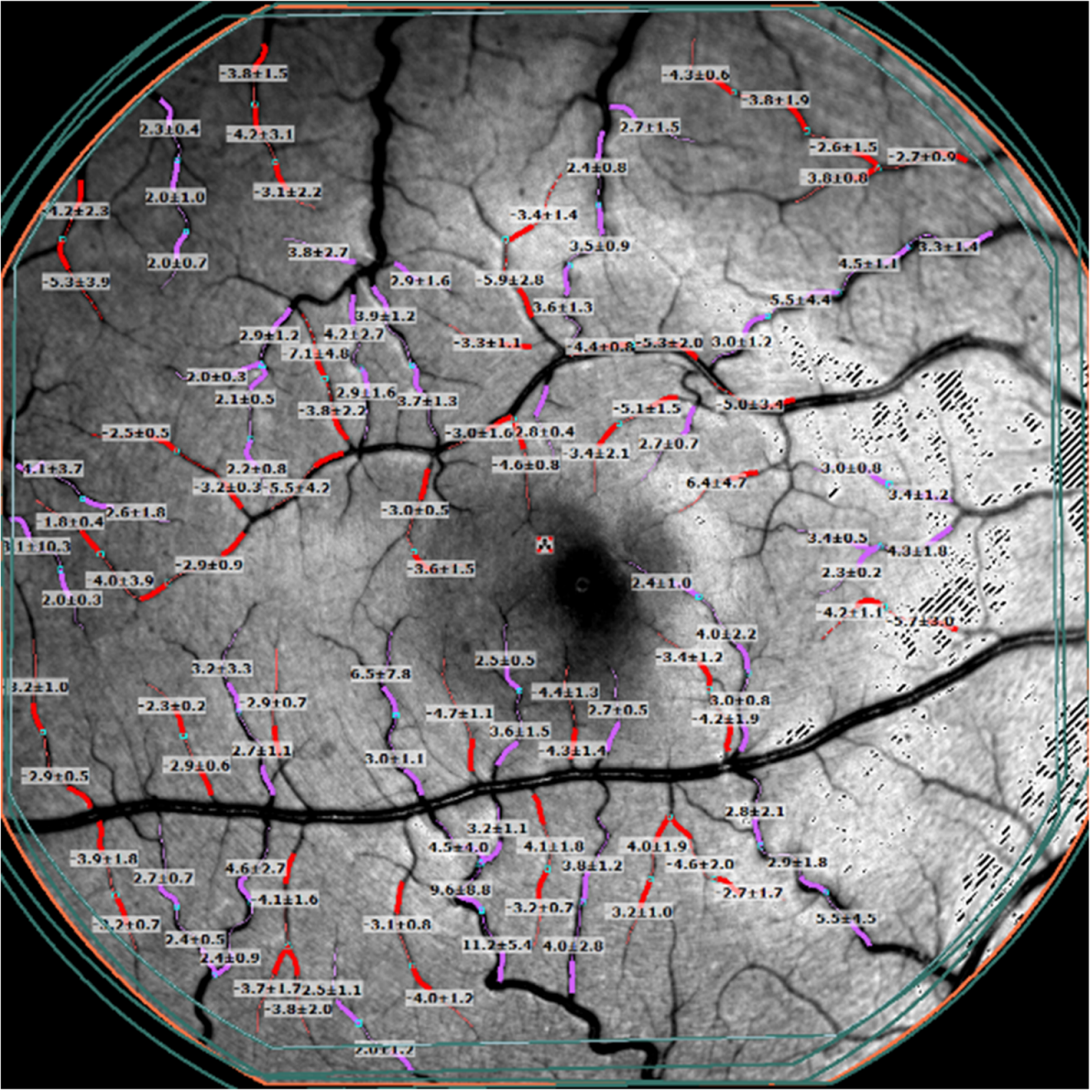
RFI image of the retina with overlaid blood flow velocity measurements. The retinal microvasculature network, centered on the fovea, was imaged to create at least 4 series of at least 4 sequential images. Secondary and tertiary branches in the visible region of the vessel map were manually outlined in segments of 60 to 90 pixels. The arteriolar (red) and venular (pink) vessel segments drawn on the vessel map marked the locations where the automated software detected blood flow. A blood flow velocity (BFV) measurement in mm/s was calculated for each corresponding vessel segment

**Fig. 2:**
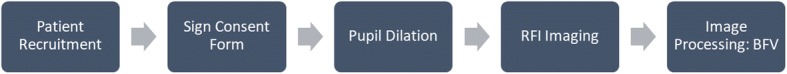
Study Procedure Flow Chart. Healthy controls and multiple sclerosis patients (with or without optic neuritis) were recruited according to inclusion and exclusion criteria. Written informed consent was obtained from all study participants. After pupillary dilation, subjects were imaged with the RFI. Processing of the resulting images yielded the arteriolar and venular blood flow velocities (BFVs)

RFI (Optical Imaging Ltd., Rehovot, Israel) was performed as described in detail elsewhere [23]. Briefly, RFI is an FDA approved advanced ophthalmic multimodal imaging modality, which measures large and small retinal vessel BVFs [23]. The device is adapted from a fundus camera with a large capacity camera (60 Hz, 1024 × 1024 pixels digital camera) and stroboscopic light source. RFI employs robust image processing software that is used to automatically derive BFVs. RFI uses red blood cell hemoglobin as an intrinsic motion contrast agent for non-invasively measuring the velocity of red blood cells in retinal vessels. During RFI acquisition, participants are asked to relax for 15 min before imaging, and pupils are dilated with 1% topical tropicamide. The scan protocol utilized was the standard RFI scan protocol [7, 26, 27]. The macula, centered on the fovea, was imaged at 35 degrees with a field of view of 7.3 × 7.3 mm^2^. Multiple image series (4–8 series) were obtained as recommended [7, 27]. The secondary and tertiary vessel segments of the retina were manually outlined, allowing the BFV measurements to be automatically calculated by the proprietary software for the arterioles and venules (Fig. 1) [23, 24]. The study procedures are outlined in the flow chart in Fig. 2.

### Visual function testing

High-contrast (100%) visual acuity at 4 m and low-contrast letter acuity (2.5% and 1.25%) at 2 m were assessed using retro-illuminated Early Treatment Diabetic Retinopathy Study and Sloan letter charts (Precision Vision, Lasalle, IL), respectively. The number of correctly identified letters was recorded for each eye (maximum score of 70 letters per chart). Visual assessments were performed in a darkened room by trained technicians using standard testing protocols, with participants using their habitual distance corrective lenses [28].

### Statistical analysis

Sample size was calculated using a software program (G*Power, Ver. 3.1.9) developed by Faul [29]. Based on the BFVs of the HC and MS groups, a sample size of 18 eyes in each group would be enough to detect the true difference (0.6 mm/s) of the blood flow velocity in the MSNON group with a detection power of 0.9. The rest of the statistical analyses were performed using Stata (version 15; StataCorp LP, College Station, TX). The Shapiro-Wilk test was used to assess the normality of distributions. The Wilcoxon rank-sum test was performed for comparisons between HC and MS groups for age and also between MS subtypes for age, disease duration, and letter acuity scores (100% high contrast, 2.5% low contrast or 1.25% low contrast letter acuity). Comparisons between HC and MS groups and between MS subtypes were assessed using the chi-squared (*χ*^2^) test for sex and race. Paired t-test was used to analyze differences in BFVs between OD and OS in HCs, and the Pearson linear correlation coefficients were used to determine correlations in inter-eye BFVs. Bland-Altman mean difference plots (illustrating the average difference between measures) were used to assess agreement and potential bias in BFVs between OD and OS in HCs across the ranges of BFV values. Pearson’s correlations were used to assess relationships between BFVs, sex, age, and race in HCs and MS, as well as disease duration and letter acuity scores in MS. Differences in average BFV between the HC and MS groups were analyzed using mixed-effects linear regression with random intercepts adjusting for age and sex. Since both eyes of HCs underwent RFI, models in which both HC eyes were utilized also accounted for within-subject inter-eye correlations. Mixed-effects linear regression adjusting for age, sex, and disease duration were also used to determine differences in BFVs between MSON and MSNON eyes. We defined significance as *P* ≤ 0.05.

## Results

The 20 HCs, consisting of 14 females and 6 males, underwent imaging with RFI of OD and OS. The average age of the HCs was 29.8 ± 10.3 years. The 38 people with MS (27 females and 11 males) were an average of 36.4 ± 10.4 years of age. The MS cohort comprised of a total of 25 patients without ON (MSNON) and 13 patients with ON (MSON) history. Baseline demographics and characteristics of the study cohorts are summarized in Table 1. There was no difference in sex among the HC, MSNON, and MSON cohorts. On average, the overall MS cohort was older than the HC cohort (*P* = 0.02). In regard to the MS cohort, disease duration was not significantly different between the MSNON and MSON groups (Table 1).

**Table 1:** Demographics and characteristics of the study cohorts

		Controls	All MS	MSNON	MSON	P value			
		(40, eyes)^a^	(38, eyes)	(25, eyes)	(13, eyes)	(All MS vs. HC)	(MSNON vs. HC)	(MSON vs. HC)	(MSON vs. MSNON)
Age, yr.; mean (SD)		29.8 (10.3)	36.4 (10.4)	36.9 (11.2)	35.6 (9.1)	**0.002** ^**b**^	**0.009** ^**b**^	**0.013** ^**b**^	0.7^b^
Sex; Female n (%)		14 (70%)	27 (71%)	18 (72%)	9 (69%)	0.92^c^	0.86^c^	0.96^c^	0.86^c^
Disease Duration, yr.; median (Q1–3)		–	7 (3,15)	6 (3,13)	8 (4,15)	–	–	–	0.44^b^
Race; Caucasian n (%)		11 (55%)	23 (65%)	15 (60%)	8 (62%)	0.13^c^	0.27^c^	0.54^c^	1.00^c^
Contrast Letter Acuity; mean (SD)	100%	–	55.9 (11.1)	57.0 (10.3)	54.0 (12.7)	–	–	–	0.63^b^
	2.5%	–	26.6 (13.7)	26.6 (13.9)	26.5 (13.8)	–	–	–	0.87^b^
	1.25%	–	12.2 (11.5)	13.5 (12.2)	9.8 (10.2)	–	–	–	0.34^b^

*HCs* = healthy controls; *MS* = multiple sclerosis; *MSNON* = MS without optic neuritis; *MSON* = MS with optic neuritis; *yr* = year; *SD* = standard deviation; *Q1* = first quartile; *Q3* = third quartile

^a^both eyes of HCs were included in the study (HC subjects, *n* = 20) while only one eye of each patient with MS was included (MS patients, n = MS eyes)

^b^Wilcoxon rank-sum test

^c^Chi-squared test

*P* ≤ 0.05 indicates significance

**Fig. 3:**
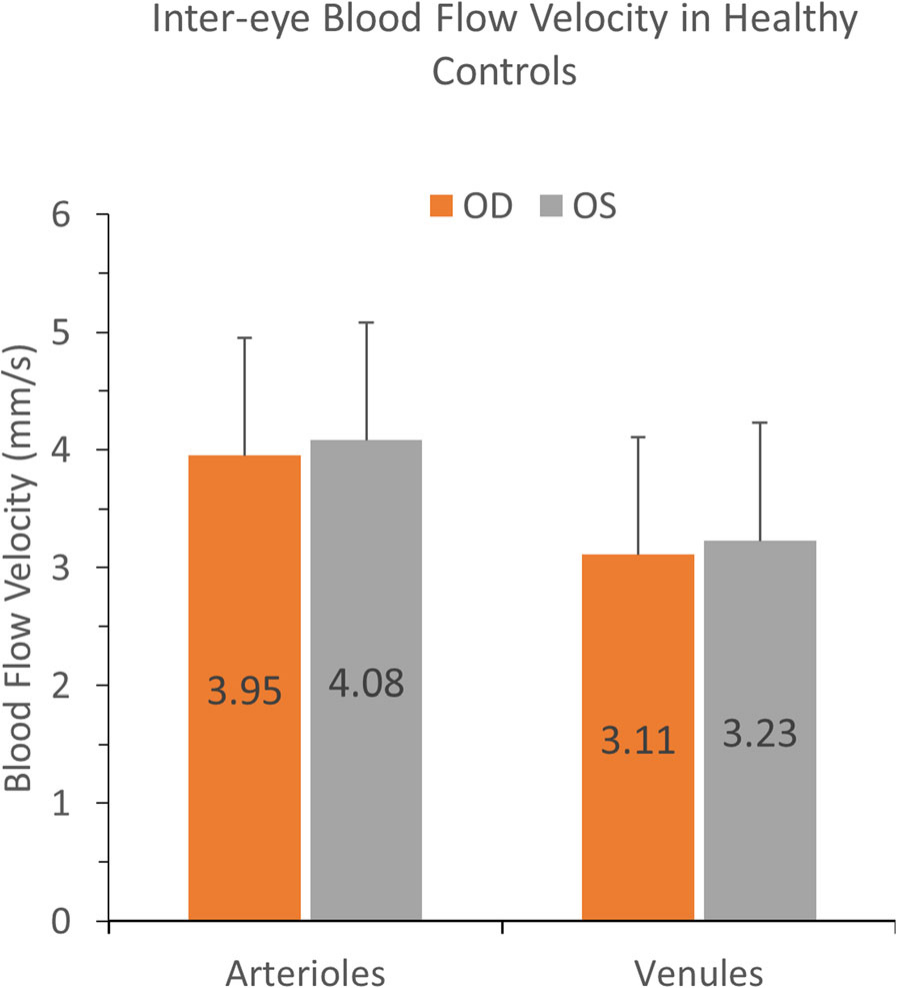
Blood flow velocities (BFVs) between OD and OS. BFVs were measured using RFI in a field of view of 35 degrees in the healthy control (HC) group (*N* = 20). BFVs were not significantly different between OD and OS for arterioles (*P* = 0.10) and venules (*P* = 0.06) in the HC group. Bars = standard deviations

**Fig. 4:**
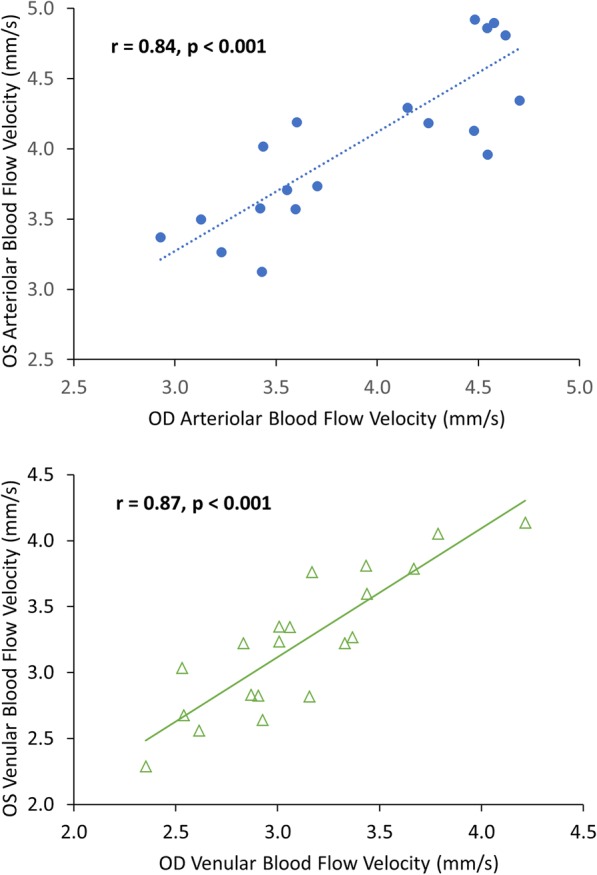
Relations of blood flow velocities (BFVs) between OD and OS in healthy controls (HCs). Both BFVs in arterioles and venules were measured in OD and OS of the HC cohort (N = 20, 40 eyes). Strong inter-eyes correlations in BFVs were found in the arterioles (top panel, *r* = 0.84, *P* < 0.001) and venules (bottom panel, *r* = 0.87, P < 0.001)

**Fig. 5:**
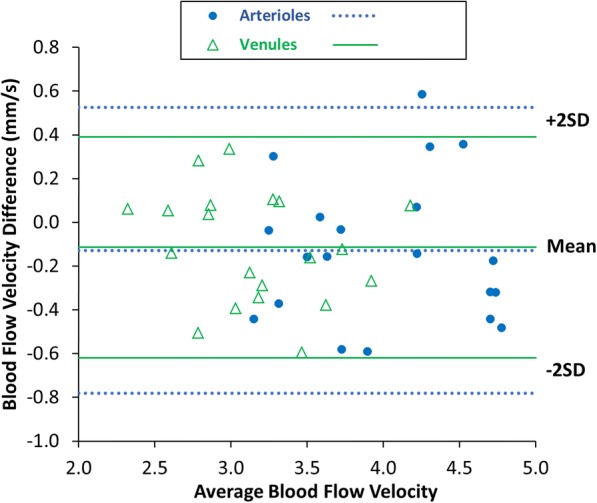
Bland Altman mean difference plot to compare blood flow velocities (BFVs) between OD and OS in the healthy control group (N = 20, 40 eyes). The difference plot shows good agreement between the OD and OS BFVs with no obvious bias

Overall, HCs had an average arteriolar BFV of 4.01 ± 0.84 mm/s and average venular BFV of 3.17 ± 0.69 mm/s (Table 2). In OD, HCs had an average arteriolar BFV of 3.95 ± 0.59 mm/s and venular BFV of 3.11 ± 0.46 mm/s. In OS, HCs had an average arteriolar BFV of 4.08 ± 0.60 mm/s and venular BFV of 3.23 ± 0.52 mm/s (Table 2). On average, BFVs in the HC cohort were moderately associated with female sex, with BFVs being faster in female eyes than male eyes in both arterioles (*r* = 0.48, *P* = 0.002, Table 3) and venules (*r* = 0.47, P = 0.002, Table 3). BFVs in HC eyes were not significantly different between OD and OS for arterioles (Fig. 3, *P* = 0.10) or for venules (Fig. 3, *P* = 0.06), and also exhibited strong inter-eye correlations in arterioles (Fig. 4, *r* = 0.84, *P* < 0.001) and venules (Fig. 4, *r* = 0.87, *P* < 0.001). Furthermore, mean difference Bland-Altman plots demonstrated good agreement in BFVs between OD and OS in HC eyes without obvious systematic bias across the ranges of BFV values (Fig. 5).

**Table 2:** Comparisons of blood flow velocities

	HC - OD	HC - OS	All MS eyes	MSNON eyes	MSON eyes	P value				
	(20, eyes)	(20, eyes)	(38, eyes)	(25, eyes)	(13, eyes)	(OD vs. OS in HCs)	(All MS vs. HCs)	(MSNON vs. HCs)	(MSON vs. HCs)	(MSON vs. MSNON)
Arterioles, mm/s; mean (SD)	3.95 (0.59)	4.08 (0.60)	3.51 (0.81)	3.48 (0.88)	3.59 (0.69)	0.1^a^	**0.01** ^**b**^	**0.009** ^**b**^	**0.046** ^**b**^	0.42^b^
Venules, mm/s; mean (SD)	3.11 (0.46)	3.23 (0.52)	2.76 (0.50)	2.75 (0.53)	2.80 (0.45)	0.064^a^	**0.003** ^**b**^	**0.005** ^**b**^	**0.048** ^**b**^	0.48^b^

*HC* = healthy control; *OD* = right; *OS* = left; *MS* = multiple sclerosis; *MSNON* = multiple sclerosis without optic neuritis; *MSON* = multiple sclerosis with optic neuritis; *SD* = standard deviation

^a^Paired t-test

^b^Mixed-effects linear regression adjusted for age, sex, and within-subject inter-eye correlations in HCs (as well as disease duration between the MS groups)

*P* ≤ 0.05 indicates significance

**Table 3:** Blood flow velocity associations in healthy controls (HCs) and multiple sclerosis (MS)

	HCs, r value (*P* value)		MS, r value (P value)	
	Arterioles	Venules	Arterioles	Venules
Age	0.21 (0.19)	−0.13 (0.43)	0.04 (0.83)	0.02 (0.92)
Sex	−0.48 (**0.002**)	−0.47 (**0.002**)	− 0.03 (0.88)	−0.2 (0.22)
Disease duration	–	–	−0.13 (0.44)	−0.16 (0.34)
Race	0.10 (0.54)	−0.26 (0.11)	0.14 (0.42)	0.13 (0.43)

*HC =* healthy control; *MS* = multiple sclerosis

*P* ≤ 0.05 indicates significance

**Fig. 6:**
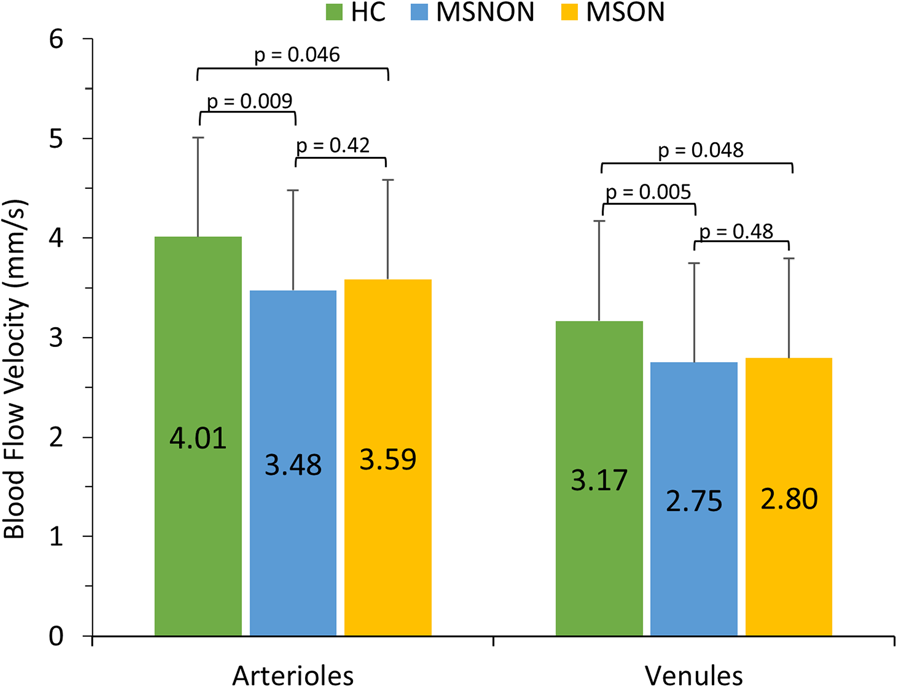
Blood flow velocities (BFVs) in multiple sclerosis (MS) eyes as compared to healthy control (HC) eyes. BFVs in the multiple sclerosis with optic neuritis (MSON) and multiple sclerosis without optic neuritis (MSNON) eyes were significantly lower than the average BFVs in HC eyes in arterioles and venules. BFVs in MS patients were derived from one eye of each participant. Average BFVs in HC eyes were derived from both eyes of participants. Bars = standard deviations

Overall, MS eyes collectively exhibited average arteriolar BFVs of 3.51 ± 0.81 mm/s and venular BFVs of 2.76 ± 0.50 mm/s. In MSNON eyes, arteriolar BFV was 3.48 ± 0.88 mm/s and venular BFV was 2.75 ± 0.53 mm/s. In MSON eyes, arteriolar BFV was 3.59 ± 0.69 mm/s and venular BFV was 2.80 ± 0.45 mm/s. BFVs in MS eyes were not significantly correlated with age, sex (unlike HCs), race, or disease duration (Table 3). Accounting for within-subject inter-eye correlations in HC eyes and adjusting for age and sex, arteriolar and venular BFVs in MS eyes were significantly lower (arterioles: *P* = 0.01; venules: *P* = 0.003) than in HC eyes (Fig. 6, Table 2). In subgroup analyses, MSNON eyes exhibited lower arteriolar and venular BFVs than HC eyes (arterioles: *P* = 0.009; venules: *P* = 0.005, Fig. 6, Table 2). Similarly, as compared to HC eyes, arteriolar and venular BFVs in MSON eyes were significantly lower (arterioles: *P* = 0.046; venules: *P* = 0.048, Fig. 6, Table 2). Adjusting for age, sex, and disease duration, significant differences in BFVs were not noted between MSON and MSNON eyes for arterioles (*P* = 0.42) or venules (*P* = 0.48). In relation to visual function measurements, arteriolar BFVs in MSON eyes were significantly correlated with 2.5% low contrast letter-acuity scores (*r* = 0.60, *P* = 0.0321). Otherwise, no significant correlations were detected in arteriolar or venular BFVs in MSON and MSNON eyes with 100% high-contrast, or 2.5% or 1.25% low contrast letter acuity scores (results not shown).

## Discussion

Although retinal BFV, as a measure of the retinal microcirculation and a potential window into the cerebral microcirculation, has provided useful information across disease states both of academic and clinical utility, measurement variability and inter-eye variability of this measurement has remained a major concern, potentially limiting and hindering the interpretation of prior studies utilizing retinal BFVs. The current study addresses this gap, assessing inter-eye variability of BFV as determined using RFI in the 35-degree field of view (FOV). Inter-eye variability was previously investigated using a 20-degree FOV, in which relatively weaker correlations in arteriolar and venular inter-eye BFVs of 0.69 and 0.62, respectively, were observed [27]. BFV correlations in our study between OD and OS appear to be much stronger as compared to these previously reported results and may relate to the different FOVs. The larger FOV at 35 degrees may theoretically include more vessel segments because the image area is larger. Nonetheless, both studies indicate that OD and OS in HCs are correlated with approximately similar BFV measurements, thereby allowing either eye to be selected for study and comparison of BFVs. Other validations of BFV reliability include intra-visit and inter-visit variability [27]. BFVs measured with RFI have been shown to be reproducible with an average intra-visit BFV variability of 7.5% (SD, 3.7%) and average inter-visit correlation of *r* = 0.74 [27]. MS eyes have previously been shown to exhibit lower BFV, as reported by Jiang et al., who studied 17 MS patients without optic neuritis (ON) history [2]. Although a different FOV (20 degrees) was used in this study, our results using the 35 degree FOV are in agreement with this previous report [2]. Moreover, MS eyes (with or without ON history) have previously been shown to exhibit a decrease in extraocular blood flow velocity in comparison to healthy controls [30]. Similarly, our study findings demonstrate a reduction in retinal BFV in MS eyes, regardless of a history of ON, raising the possibility that reductions in microvascular blood flow could be a global occurrence as part of the MS disease process, although this requires further exploration and definitive confirmation.

The ON sample size in the current study is small and therefore findings in this cohort need to be interpreted with caution, including the lack of difference observed in BFVs between MSON and MSNON eyes. Indeed, the reduction in arteriolar and venular BFVs in MSON eyes was only borderline lower than in HC eyes, again likely related to less power in the MSON cohort, as compared to the larger MSNON cohort. In general, ON in MS eyes causes axonal loss within the optic nerve, resulting in thinning of the retinal nerve fiber layer (RNFL) and the combined ganglion cell and inner plexiform layer (GCIPL) [31–33]. Therefore, it is plausible that the decreased BFV in MSON eyes (as compared to HC eyes) could simply be secondary to retinal neurodegeneration and its related decrease in metabolic demand. However, we did not find a significant difference in BFV between MSON and MSNON eyes, with both eyes exhibiting similar overall reductions in BFVs, thereby potentially arguing against this hypothesis. The lack of difference in BFVs between MSON and MSNON eyes could relate to bias in an excess of more subclinical optic neuropathy in the MSNON cohort included in this study. Optic neuropathy in MS may be overt (as in clinical optic neuritis) or occult (as in subclinical optic neuropathy), with optic nerve affliction being virtually ubiquitous as part of the MS disease process [34, 35]. Nevertheless, this raises the possibility that microcirculation aberrations may be more widespread in MS than previously thought. Indeed, a relationship between RFI-derived BFVs and peripapillary RNFL thickness was not found in a prior study of RFI in MS eyes potentially supporting this hypothesis, although further work is necessary in the future to explore this possibility [2]. Chronic cerebrospinal venous insufficiency (CCSVI) has been proposed as a potential pathobiologic mechanism underlying MS and could potentially provide an explanation for global hemodynamic alterations in the retina, since increased severity in CCSVI has been suggested to be associated with decreased cerebral blood flow (CBF) [36–38]. However, CBF was not measured in the present study, and there has been an abundance of studies to date largely disbanding the theory that CCSVI plays a role in MS [39–47]. Alternatively, these findings may also suggest the presence of auto-regulation mechanisms compensating for and adjusting BFVs in eyes with a history of ON. Of course, any differences or lack thereof noted between MSON and MSNON eyes in the current study may simply be indicative of inadequate sample size. It is worth noting however, that MSON eyes showed a positive, significant correlation between arteriolar BFV and 2.5% low contrast letter acuity scores. This relationship suggests that a relatively higher BFV could be physiologically relevant for visual function in patients with MS but requires further exploration and validation.

Our study has a number of limitations. We did not measure BFV in both eyes of MS patients since the imaging procedure requires pupillary dilation with bilateral scanning potentially leading to additional time and discomfort. However, as noted in previous studies [2, 7, 23], the measurement of one eye of participants may be sufficient to demonstrate alterations in the retinal microcirculation. Moreover, the measurement of BFVs was not fully automated since the vessels were manually traced and sometimes designated as arterioles or venules by the operator [7, 27]. Furthermore, we did not acquire RFI scans with both the 35 and 20 degree FOV, which precludes direct comparison of our study findings to those in which the 20 degree FOV was utilized [27]. Finally, the sample sizes of the MSON and MSNON cohorts were small, in particular being slightly underpowered in the MSON cohort based on the power calculations performed.

## Conclusions

Inter-eye BFVs are strongly correlated without significant differences in HC eyes. Our study findings also support prior observations of reduced arteriolar and venular BFVs in MS eyes. Interestingly, this seems to be the case regardless of ON history, thereby suggesting that microvascular alterations in MS may be widespread. Although we did not find differences in BFVs between MSON and MSNON eyes, larger and longitudinal studies are required in the future to more definitively assess this and to relate results to established retinal biomarkers of neurodegeneration.

## References

[1] Jokinen H, Lipsanen J, Schmidt R, Fazekas F, Gouw AA, van der Flier WM (2012). Brain atrophy accelerates cognitive decline in cerebral small vessel disease: the LADIS study. Neurology.

[2] Jiang H, Delagao S, Tan J, Liu C, Rammohan KW, DeBuc DC (2016). Impaired retinal microcirculation in multiple sclerosis. Mult Scler.

[3] Burgansky-Eliash Z, Barak A, Barash H, Nelson DA, Pupko O, Lowenstein A (2012). Increased retinal blood flow velocity in patients with early diabetes mellitus. Retina.

[4] Gutfreund S, Izkhakov E, Pokroy R, Yaron M, Yeshua H, Burgansky-Eliash Z (2013). Retinal blood flow velocity in metabolic syndrome. Graefes Arch Clin Exp Ophthalmol.

[5] Burgansky-Eliash Z, Barash H, Nelson D, Grinvald A, Sorkin A, Loewenstein A (2014). Retinal blood flow velocity in patients with age-related macular degeneration. Curr Eye Res.

[6] Landa G, Amde W, Haileselassie Y, Rosen RB (2011). Cilioretinal arteries in diabetic eyes are associated with increased retinal blood flow velocity and occurrence of diabetic macular edema. Retina.

[7] Chhablani J, Bartsch DU, Cheng L, Gomez L, Alshareef RA, Rezeq SS (2013). Segmental reproducibility of retinal blood flow velocity measurements using retinal function imager. Graefes Arch Clin Exp Ophthalmol.

[8] Minagar A, Jy W, Jimenez JJ, Alexander JS (2006). Multiple sclerosis as a vascular disease. Neurol Res.

[9] Wakefield A J, More L J, Difford J, McLaughlin J E (1994). Immunohistochemical study of vascular injury in acute multiple sclerosis. Journal of Clinical Pathology.

[10] Cramer SP, Modvig S, Simonsen HJ, Frederiksen JL, Larsson HB (2015). Permeability of the blood-brain barrier predicts conversion from optic neuritis to multiple sclerosis. Brain.

[11] Law M, Saindane AM, Ge Y, Babb JS, Johnson G, Mannon LJ (2004). Microvascular abnormality in relapsing-remitting multiple sclerosis: perfusion MR imaging findings in normal-appearing white matter. Radiology.

[12] Saindane AM, Law M, Ge Y, Johnson G, Babb JS, Grossman RI (2007). Correlation of diffusion tensor and dynamic perfusion MR imaging metrics in normal-appearing corpus callosum: support for primary hypoperfusion in multiple sclerosis. AJNR Am J Neuroradiol.

[13] Inglese M, Adhya S, Johnson G, Babb JS, Miles L, Jaggi H (2008). Perfusion magnetic resonance imaging correlates of neuropsychological impairment in multiple sclerosis. J Cereb Blood Flow Metab.

[14] Adhya Sumita, Johnson Glyn, Herbert Joseph, Jaggi Hina, Babb James S., Grossman Robert I., Inglese Matilde (2006). Pattern of hemodynamic impairment in multiple sclerosis: Dynamic susceptibility contrast perfusion MR imaging at 3.0 T. NeuroImage.

[15] Varga AW, Johnson G, Babb JS, Herbert J, Grossman RI, Inglese M (2009). White matter hemodynamic abnormalities precede sub-cortical gray matter changes in multiple sclerosis. J Neurol Sci.

[16] Brooks DJ, Beaney RP, Powell M, Leenders KL, Crockard HA, Thomas DG (1986). Studies on cerebral oxygen metabolism, blood flow, and blood volume, in patients with hydrocephalus before and after surgical decompression, using positron emission tomography. Brain.

[17] Trapp BD, Stys PK (2009). Virtual hypoxia and chronic necrosis of demyelinated axons in multiple sclerosis. Lancet Neurol.

[18] Juurlink BH (1998). The multiple sclerosis lesion: initiated by a localized hypoperfusion in a central nervous system where mechanisms allowing leukocyte infiltration are readily upregulated?. Med Hypotheses.

[19] D'haeseleer M, Cambron M, Vanopdenbosch L, De Keyser J (2011). Vascular aspects of multiple sclerosis. Lancet Neurol.

[20] Feucht N, Maier M, Lepennetier G, Pettenkofer M, Wetzlmair C, Daltrozzo T, et al. Optical coherence tomography angiography indicates associations of the retinal vascular network and disease activity in multiple sclerosis. Mult Scler. 2018:1352458517750009. 10.1177/1352458517750009. 10.1177/1352458517750009 29303033

[21] Lanzillo R, Cennamo G, Criscuolo C, Carotenuto A, Velotti N, Sparnelli F, et al. Optical coherence tomography angiography retinal vascular network assessment in multiple sclerosis. Mult Scler. 2017:1352458517729463. 10.1177/1352458517729463. 10.1177/1352458517729463 28933233

[22] Polman CH, Reingold SC, Banwell B, Clanet M, Cohen JA, Filippi M (2011). Diagnostic criteria for multiple sclerosis: 2010 revisions to the McDonald criteria. Ann Neurol.

[23] Wang L, Jiang H, Grinvald A, Jayadev C, Wang J (2018). A mini review of clinical and research applications of the retinal function imager. Curr Eye Res.

[24] Ganekal S (2013). Retinal functional imager (RFI): non-invasive functional imaging of the retina. Nepal J Ophthalmol.

[25] Landa G, Jangi AA, Garcia PM, Rosen RB (2012). Initial report of quantification of retinal blood flow velocity in normal human subjects using the retinal functional imager (RFI). Int Ophthalmol.

[26] GRINVALD A, BONHOEFFER T, VANZETTA I, POLLACK A, ALONI E, OFRI R, NELSON D (2004). High-resolution functional optical imaging: from the neocortex to the eye. Ophthalmology Clinics of North America.

[27] Burgansky-Eliash Z, Lowenstein A, Neuderfer M, Kesler A, Barash H, Nelson DA (2013). The correlation between retinal blood flow velocity measured by the retinal function imager and various physiological parameters. Ophthalmic Surg Lasers Imaging Retina.

[28] Talman LS, Bisker ER, Sackel DJ, Long DA, Galetta KM, Ratchford JN (2010). Longitudinal study of vision and retinal nerve fiber layer thickness in multiple sclerosis. Ann Neurol.

[29] Faul F, Erdfelder E, Lang AG, Buchner A (2007). G*Power 3: a flexible statistical power analysis program for the social, behavioral, and biomedical sciences. Behav Res Methods.

[30] Pache M, Kaiser HJ, Akhalbedashvili N, Lienert C, Dubler B, Kappos L (2003). Extraocular blood flow and endothelin-1 plasma levels in patients with multiple sclerosis. Eur Neurol.

[31] Bourdette DN, Cohen JA (2014). Venous angioplasty for "CCSVI" in multiple sclerosis: ending a therapeutic misadventure. Neurology.

[32] Petzold A, Wattjes MP, Costello F, Flores-Rivera J, Fraser CL, Fujihara K (2014). The investigation of acute optic neuritis: a review and proposed protocol. Nat Rev Neurol.

[33] Costello F, Hodge W, Pan YI, Eggenberger E, Coupland S, Kardon RH (2008). Tracking retinal nerve fiber layer loss after optic neuritis: a prospective study using optical coherence tomography. Mult Scler.

[34] Gundogan FC, Tas A, Altun S, Oz O, Erdem U, Sobaci G (2013). Color vision versus pattern visual evoked potentials in the assessment of subclinical optic pathway involvement in multiple sclerosis. Indian J Ophthalmol.

[35] Engell Tine, Trojaborg Werner, Raun Niels Erik (2009). Subclinical optic neuropathy in multiple sclerosis. Acta Ophthalmologica.

[36] Zamboni P, Menegatti E, Weinstock-Guttman B, Dwyer MG, Schirda CV, Malagoni AM (2011). Hypoperfusion of brain parenchyma is associated with the severity of chronic cerebrospinal venous insufficiency in patients with multiple sclerosis: a cross-sectional preliminary report. BMC Med.

[37] Ciciarello F, Mandolesi S, Galeandro AI, Marceca A, Rossi M, Fedele F (2014). Age-related vascular differences among patients suffering from multiple sclerosis. Curr Neurovasc Res.

[38] Ciccone MM, Galeandro AI, Scicchitano P, Zito A, Gesualdo M, Sassara M (2012). Multigate quality Doppler profiles and morphological/hemodynamic alterations in multiple sclerosis patients. Curr Neurovasc Res.

[39] Costello F, Modi J, Lautner D, Bhayana D, Scott JN, Davenport WJ (2014). Validity of the diagnostic criteria for chronic cerebrospinal venous insufficiency and association with multiple sclerosis. CMAJ.

[40] Brod SA, Kramer LA, Cohen AM, Barreto AD, Bui TT, Jemelka JR (2013). Chronic cerebrospinal venous insufficiency: masked multimodal imaging assessment. Mult Scler.

[41] Ghezzi A, Annovazzi P, Amato MP, Capello E, Cavalla P, Cocco E (2013). Adverse events after endovascular treatment of chronic cerebro-spinal venous insufficiency (CCSVI) in patients with multiple sclerosis. Mult Scler.

[42] Benedict RH, Weinstock-Guttmam B, Marr K, Valnarov V, Kennedy C, Carl E (2013). Chronic cerebrospinal venous insufficiency is not associated with cognitive impairment in multiple sclerosis. BMC Med.

[43] Baracchini C, Atzori M, Gallo P (2013). CCSVI and MS: no meaning, no fact. Neurol Sci.

[44] Leone C, D'Amico E, Cilia S, Nicoletti A, Di Pino L, Patti F (2013). Cognitive impairment and "invisible symptoms" are not associated with CCSVI in MS.. BMC Neurol.

[45] Van den Berg PJ, Van den Berg GB, Westerhuis LW, Visser LH (2013). Occurrence of CCSVI in patients with MS and its relationship with iron metabolism and varicose veins. Eur J Neurol.

[46] Jedynak W, Cieszanowski A (2014). Is there any relation between chronic cerebrospinal venous insufficiency and multiple sclerosis? - a critical review. Pol J Radiol.

[47] Van den Berg PJ, Visser LH (2013). The fluctuating natural course of CCSVI in MS patients and controls, a prospective follow-up. PLoS One.

